# Susceptibility Modules and Genes in Hypertrophic Cardiomyopathy by WGCNA and ceRNA Network Analysis

**DOI:** 10.3389/fcell.2021.822465

**Published:** 2022-02-01

**Authors:** Yifan Sun, Zhongbo Xiao, Yequn Chen, Duanmin Xu, Shuying Chen

**Affiliations:** Department of Cardiology, First Affiliated Hospital of Shantou University Medical College, Shantou, China

**Keywords:** competing endogenous RNA network, weighted correlation network analysis, hypertrophic cardiomyopathy, bioinformatics, IGFBP5

## Abstract

**Background:** We attempted to identify a regulatory competing endogenous RNA (ceRNA) network and a hub gene of Hypertrophic Cardiomyopathy (HCM).

**Methods:** Microarray datasets of HCM tissue were obtained from NCBI Gene Expression Omnibus (GEO) database. The R package “limma” was used to identify differentially expressed genes. Online search databases were utilized to match the relation among differentially expressed long non-coding RNAs (lncRNAs), microRNAs (miRNAs) and mRNAs. Weighted correlation network analysis (WGCNA) was used to identify the correlations between key modules and HCM. STRING database was applied to construct PPI networks. Gene Set Enrichment Analysis (GSEA) was used to perform functional annotations and verified the hub genes.

**Results:** A total of 269 DE-lncRNAs, 63 DE-miRNAs and 879 DE-mRNAs were identified in myocardial tissues from microarray datasets GSE130036, GSE36946 and GSE36961, respectively. According to online databases, we found 1 upregulated miRNA hsa-miR-184 that was targeted by 2 downregulated lncRNAs (SNHG9, AC010980.2), potentially targeted 2 downregulated mRNAs (LRRC8A, SLC7A5). 3 downregulated miRNAs (hsa-miR-17-5p, hsa-miR-876-3p, hsa-miR-139-5p) that were targeted by 9 upregulated lncRNAs, potentially targeted 21 upregulated mRNAs. Black and blue modules significantly related to HCM were identified by WGCNA. Hub gene IGFBP5 regulated by hsa-miR-17-5p, AC007389.5, AC104667.1, and AC002511.2 was identified. GSEA indicated that IGFBP5 might involve in the synthesis of myosin complex, participate in kinesin binding, motor activity and function via the regulation of actin cytoskeleton.

**Conclusion:** The results provide a potential molecular regulatory mechanism for the diagnosis and treatment of HCM. IGFBP5 might play an important role in the progression of HCM.

## Introduction

HCM is one of the most common autosomal-dominant cardiomyopathies with a prevalence of 1:200 to 1:500 (Maron and Maron, 2013; Semsarian et al., 2015). HCM is frequently underrecognised and diagnosed late. The annualized HCM death rate is 1.4% [2]. Importantly, HCM is an important cause of sudden cardiac death in the young (Shah, 2017; Maron, 2018). HCM is also a sarcomere-associated disease. Complicated biological processes occur LncRNA Rp5 with the progression of disease, including the hypertrophy and remodeling of left ventricular, impairment of diastolic function, myocardial ischemia, obstruction of the outflow tract, and other functional changes. Aforementioned interactions causes great heterogeneity in clinical symptoms of HCM (Elliott et al., 2014). Currently, researchers believe that gene mutations play an important role in HCM (Watkins et al., 2011). Mutations in several sacromeric genes, including *MYH7*, *MYBPC3*, *TPM1*, *TNNT2* and *TNNI3*, have been identified as crucial genetic changes in 34% HCM patients (Erdmann et al., 2003; Burns et al., 2017; Geske et al., 2018). These are usually familial and hereditary cases. However, gene mutations have not been verified in approximately 70% HCM patients so the hereditary pathogenesis was unclear.

Exons with coding function account for a <2% proportion in human genes. Non-coding RNAs (ncRNAs) include long and small non-coding sequence. Long non-coding RNAs (lncRNAs) are transcripts with more than 200 nucleotides. Usually, lncRNAs play a crucial role in regulation of gene expression by RNA-RNA, RNA-DNA and RNA-protein ways in ribonucleoprotein complexes (Small and Olson, 2011; Derrien et al., 2012; Kaur et al., 2018). Previous studies demonstrate that lncRNAs are implicated in the gene regulation of several diseases, such as acute myocardial infarction, cancer and heart failure (Kornienko et al., 2013; Zhang et al., 2019; Liang et al., 2020). In HCM patients, lncRNAs are verified to be involved in vital biological processes of myocardial fibrosis, heart failure and atherosclerosis (Wang et al., 2020). MicroRNAs (miRNAs) are 17–25 nucleotides in length with function of targeting and regulating mRNA (Jansson and Lund, 2012). Mature miRNAs are directed to the 3 end of the target mRNA by base pairing, resulting in instability and translation inhibition of mRNA (Saliminejad et al., 2019). MiRNAs have been found to be involved in signaling pathways related to cancer, myocardial ischemia and diabetic cardiomyopathy (Li et al., 2020; Farooqi et al., 2021; Pedersen et al., 2021). Notably, Song et al. (2014) found 13 kinds of miRNAs related to HCM, among which miR-10a, miR-30c and miR-373 have also been corroborated by other previous studies.

In molecular biology, lncRNAs and miRNAs are important components of competing endogenous RNAs (ceRNAs). LncRNAs play a role as sponges and bind to miRNA targets, inhibiting or promoting the regulation of miRNAs on mRNAs (Salmena et al., 2011). LncRNA-miRNA-mRNA axis is an important regulatory network for cardiovascular disease. LncRNA Rp5-833A20.1 acts as a sponge to prevent miR-382-5p to target NFIA and promote atherosclerosis (Hu et al., 2015). LncRNA MIAT has been proved to be a kind of lncRNA that promotes myocardial fibrosis after acute ischemia. It is achieved via regulating MIAT/miRNA24 to target mRNA Furin and TGF-β1 (Qu et al., 2017). LncRNA ROR sponges miR-133 to cause the re-expression of ANP and BNP, leading to the exacerbation of cardiac hypertrophy eventually (Jiang et al., 2016). Guo et al. (2020) found that the ceRNA network from plasma was contributed to the genetic regulation of HCM by using bioinformatics method. In view of these studies, we hypothesized that the ceRNA network in cardiac tissue might play a regulatory role in HCM. In this study, we attempted to construct a cardiac tissue ceRNA regulatory network for HCM using human data from GEO database.

## Materials and Methods

### Microarray Data

Human HCM microarray datasets were obtained from the GEO database (http://www.ncbi.nlm.nih.gov/geo/). GSE130036, GSE36946 and GSE36961 are three datasets containing long non-coding RNA, miRNA and mRNA profiling, respectively. The expression profiles of surgical myectomy tissues were compared between HCM patients and control donors.

### Identification of Differentially Expressed Genes

After extracting the gene expression matrixes from the above microarray datasets. The R package “limma” was used to analyze the genes that were differentially expressed. FDR < 0.05 and |log2 FC| > 0.5 were the thresholds to identify differentially expressed lncRNA and mRNA. FDR < 0.05 and |log2 FC| > 0 were the thresholds of differentially expressed miRNA.

### Construction of a Candidate LncRNA-miRNA-mRNA Network

Online search databases were utilized to predict differentially expressed lncRNA and mRNA that potentially target differentially expressed miRNA. Specifically, MiRcode (http://www.mircode.org/) database was applied to predict lncRNA that could target miRNA. And the mRNA that could potentially be targeted by miRNA in all these following databases were selected: miRTarBase (http://mirtarbase.mbc.nctu.edu.tw/), miRDB (http://www.mirdb.org/), and TargetScan (http://www.targetscan.org/) databases. Here, the lncRNA-miRNA-mRNA network was constructed in Cytoscape software using the above selected genes.

### Weighted Correlation Network Analysis (WGCNA) and Functional Annotation

In order to identify clinically key modules of HCM, The R package “WGCNA” was used to construct a weighted correlation network using the mRNA expression profiling in GSE36961. Pearson coefficient was calculated to assess the weighted co-expression relationship among all genes in the network. The soft threshold was used to ensure a scale-free network. The network interconnectedness was represented by topological overlap measure. Gene modules that consist of genes with high correlations, were identified based on the hierarchical clustering method. The gene modules with significant correlations with HCM were identified as clinically key modules. The protein-protein interaction (PPI) network of clinically key modules was constructed using the Search Tool for the Retrieval of Interacting Genes (STRING, https://string-db.org/) database. Confidence score > 0.7 was set as significant. In Cytoscape software, plug-in Molecular Complex Detection (MCODE) was used to select hub clusters of significant modules and perform functional annotation.

### Identification and Functional Annotation of Clinically Hub Genes and Regulatory Network for HCM

The intersection of genes between lncRNA-miRNA-mRNA network and above clinically key modules were identified as the clinically hub genes and regulatory network of HCM. Then, Gene Set Enrichment Analysis (GSEA) was used to perform functional annotation and determine whether the hub genes showed significant, concordant differences between HCM patients and control donors.

## Results

### Differentially Expressed LncRNA, miRNA and mRNA

**FIGURE 1:**
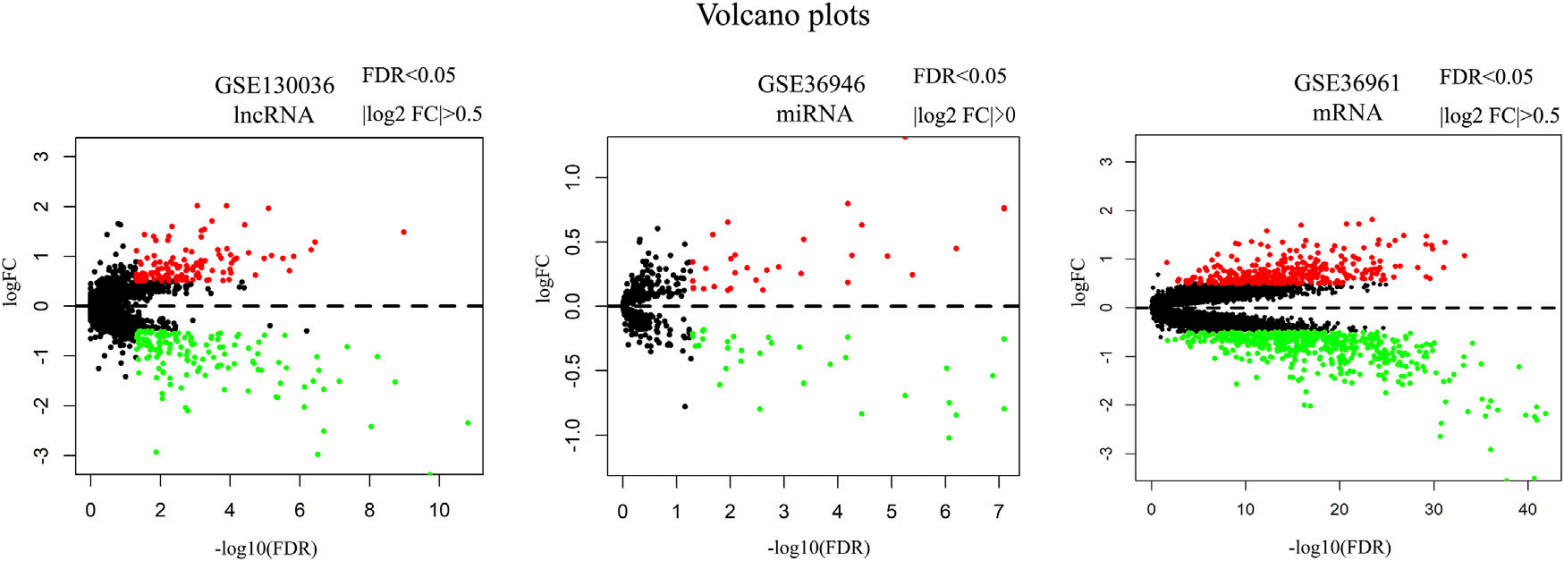
Volcano plots of differentially expressed lncRNA, miRNA and mRNA. Red dots represent upregulated genes. Green dots represent downregulated genes. Note: FC, fold change.

A total of 127 upregulated and 142 downregulated lncRNAs were found in myocardial tissues from 28 HCM patients. 31 upregulated and 32 downregulated miRNAs were identified in myocardial tissues from HCM patients. 361 upregulated and 518 downregulated miRNAs were obtained in myocardial tissues from HCM patients. Volcano maps in Figure 1 showed the differentially expressed genes in these microarray datasets.

### LncRNA-miRNA-mRNA Network

**FIGURE 2:**
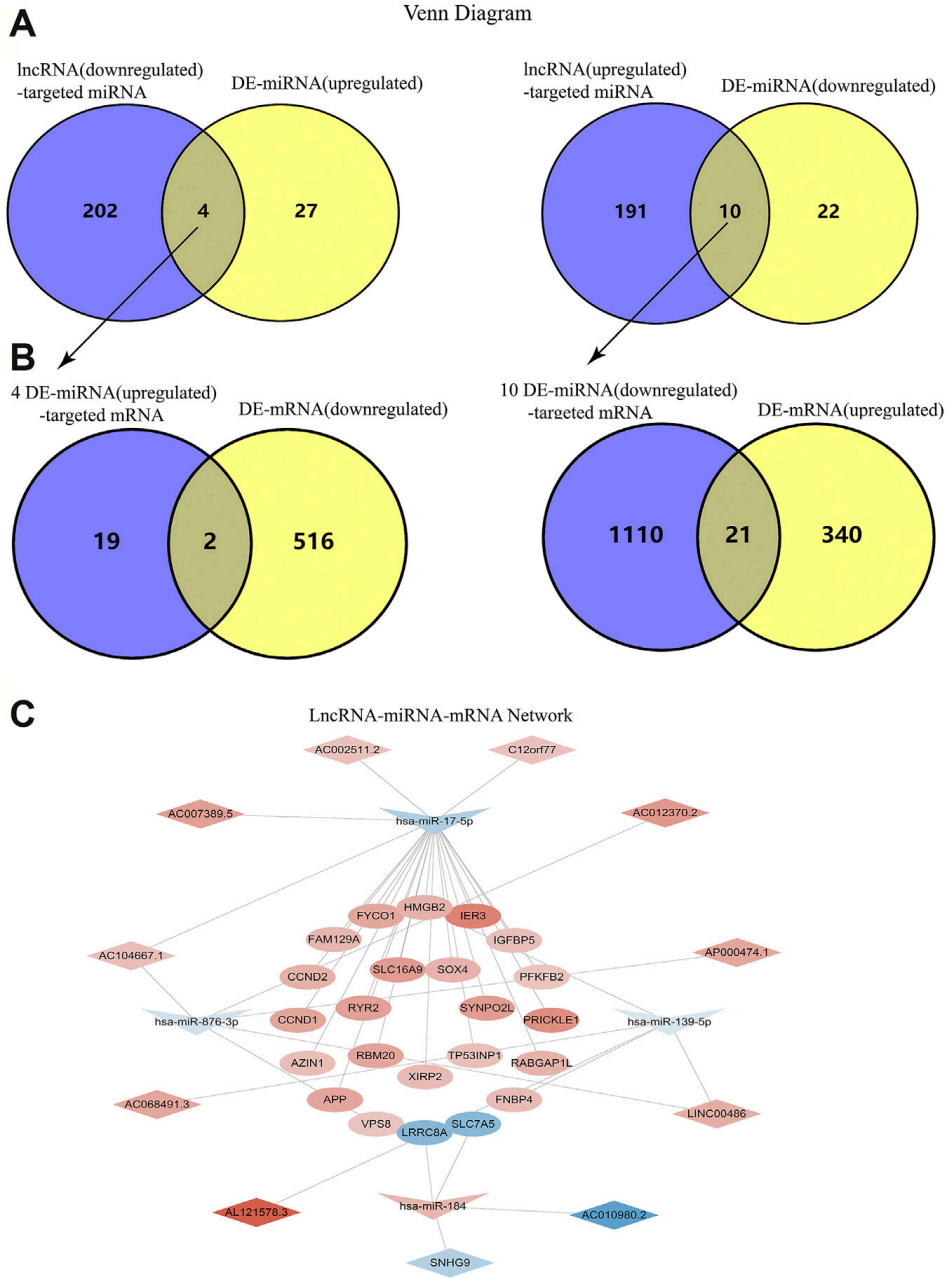
Construction of the LncRNA-miRNA-mRNA Network. **(A)** Venn diagrams of the intersection between downregulated lncRNA-targeted miRNA and upregualted miRNA, as well as the intersection between upregulated lncRNA-targeted miRNA and downregualted miRNA. **(B)** Venn diagrams of the intersection between four upregulated miRNA-targeted mRNA and downregualted mRNA, as well as the intersection between ten downregulated miRNA-targeted mRNA and upregualted mRNA. **(C)** The interaction network among lncRNA, miRNA and mRNA.

According to the information provided by several online search databases mentioned above, we found that 206 miRNAs were targeted by 142 downregulated lncRNAs while 201 miRNAs were targeted by 127 upregulated lncRNAs. After making intersections, 4 upregulated miRNAs potentially targeted by downregulated lncRNAs and 10 downregulated miRNAs potentially targeted by upregulated lncRNAs were revealed (Figure 2A). There were 21 mRNAs targeted by 4 upregulated miRNAs and 1131 mRNAs targeted by 10 downregulated miRNAs. After making intersections, two downregulated mRNAs potentially targeted by upregulated lncRNAs and 21 upregulated mRNAs potentially targeted by downregulated lncRNAs were found (Figure 2B). Hence, only 1 upregulated miRNA hsa-miR-184 that was targeted by 2 downregulated lncRNAs (SNHG9, AC010980.2), targeted 2 downregulated mRNAs (LRRC8A, SLC7A5). Three downregulated miRNAs (hsa-miR-17-5p, hsa-miR-876-3p, hsa-miR-139-5p) that were targeted by 9 upregulated lncRNAs, targeted 21 upregulated mRNAs. The details were presented in lncRNA-miRNA-mRNA network (Figure 2C).

### Clinically Key Modules and Functional Annotation

**FIGURE 3:**
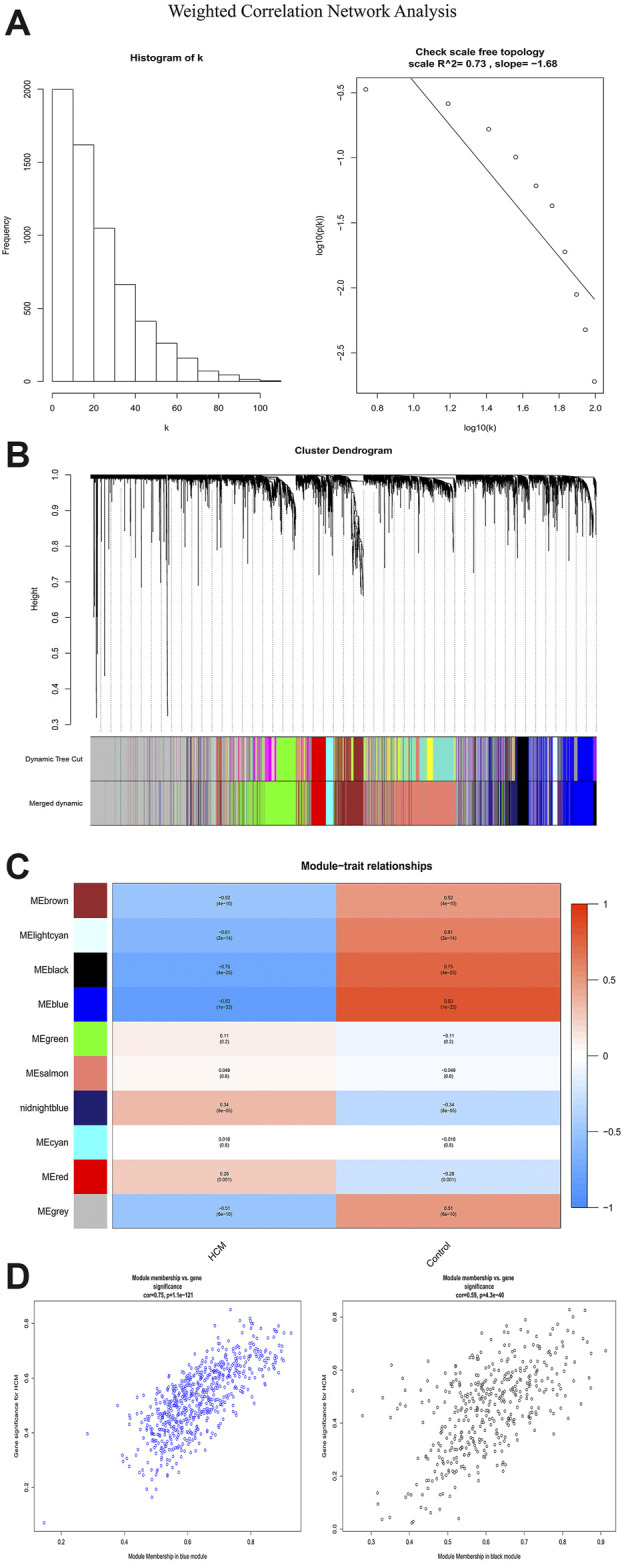
Weighted correlation network analysis. **(A)** Histogram and check scale free topology. **(B)** Cluster dendrogram of modules. **(C)** Heat map of module-HCM relationships. **(D)** Module memberships in blue and black module and their correlation with HCM. HCM, hypertrophic cardiomyopathy.

**FIGURE 4:**
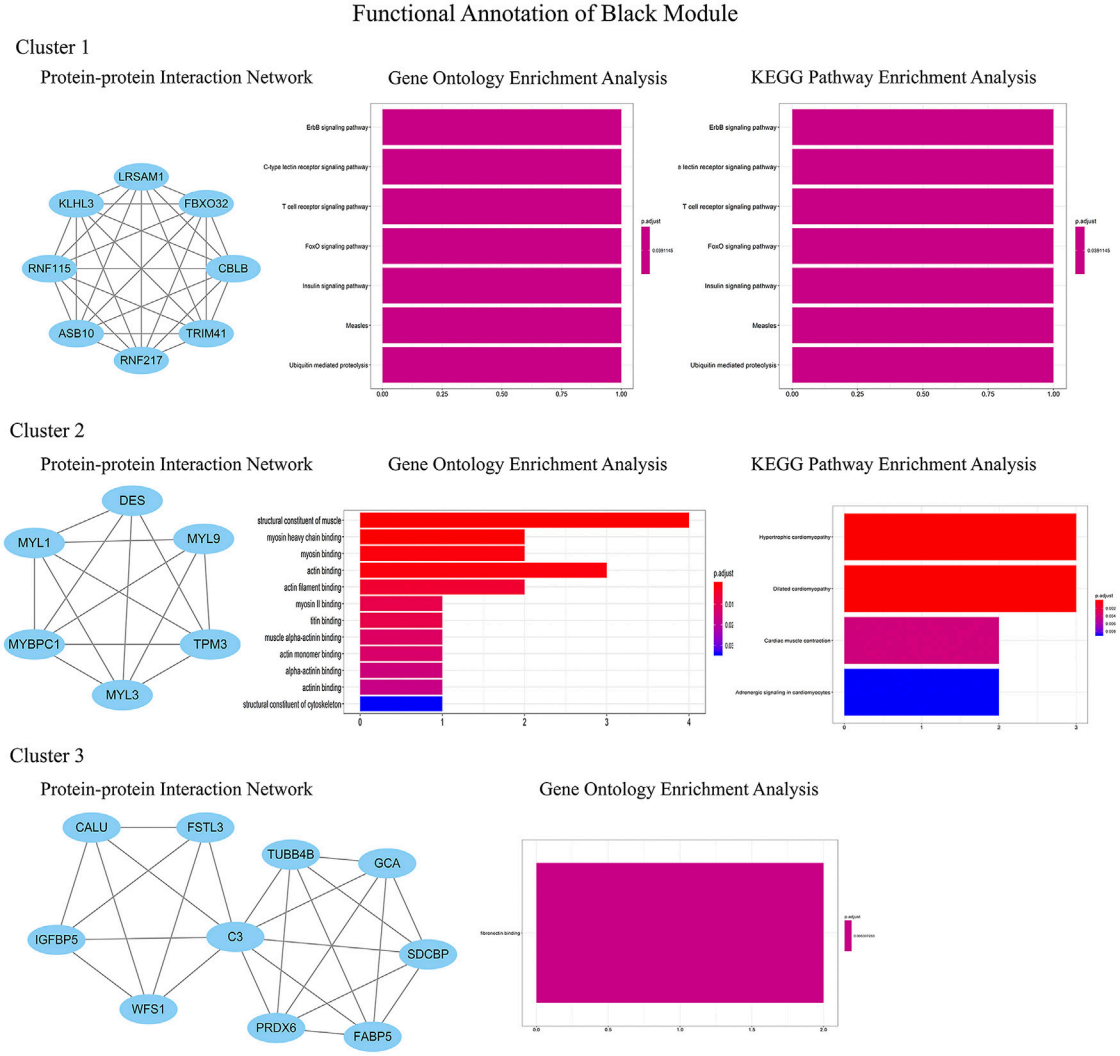
Functional annotations of three clusters in black module (including protein-protein interaction network, gene ontology enrichment analysis and KEGG pathway enrichment analysis). GO functional analysis showed that the genes in black module contributed to muscle composition, myosin, actin, and fibronectin binding, while KEGG analysis demonstrated that the genes were enriched in diastolic and contractile signaling pathways of cardiomyocytes.

**FIGURE 5:**
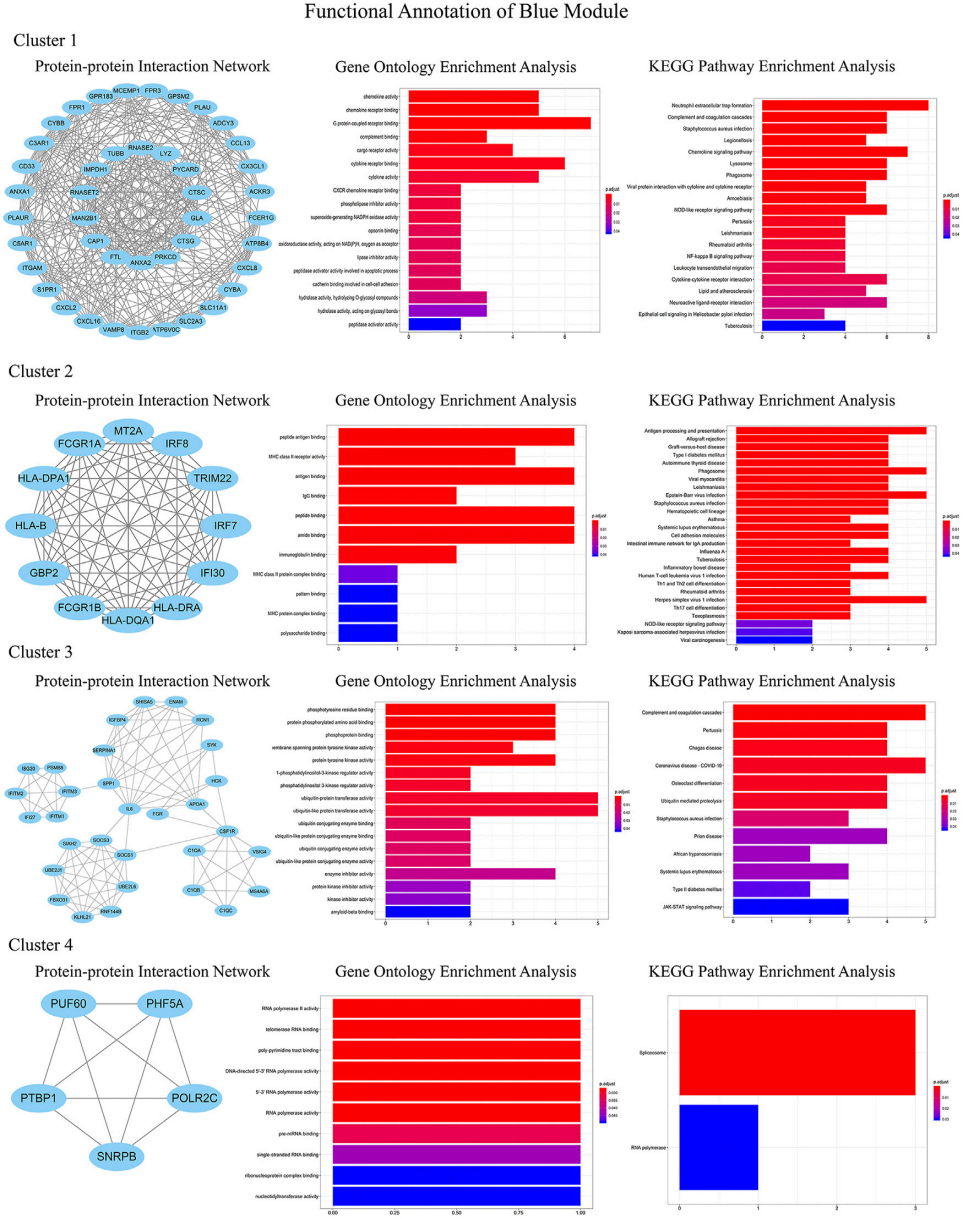
Functional annotations of four clusters in blue module (including protein-protein interaction network, gene ontology enrichment analysis and KEGG pathway enrichment analysis). Genes in blue module were involved in cytokine chemotaxis, pathogen infection, inflammatory response, antigen presentation, and atherogenesis, which may be contributed to the development of HCM.

In order to identify the relatively balanced mean connectivity and scale independence, a soft threshold of 10 was selected to construct the scale-free topology module (Supplementary File). As shown in Figure 3, a total of 10 modules were constructed, in which the black and blue modules were identified as clinically key modules. Black and blue modules were negatively related to HCM with coefficients of −0.75 (*p* = 4e-25) and −0.83 (*p* = 1e-33), respectively. 3 hub clusters in black module and 4 hub clusters in blue module were identified using plug-in MCODE in Cytoscape software. The PPI network and Functional annotation of the hub clusters in black module were listed in Figure 4. GO enrichment analysis of cluster 3 in black module indicated the importance of fibronectin binding in the biological mechanism of HCM. Likewise, The PPI network and Functional annotation of the hub clusters in blue module were listed in Figure 5.

### Clinically Hub Gene and Regulatory Network of HCM

**FIGURE 6:**
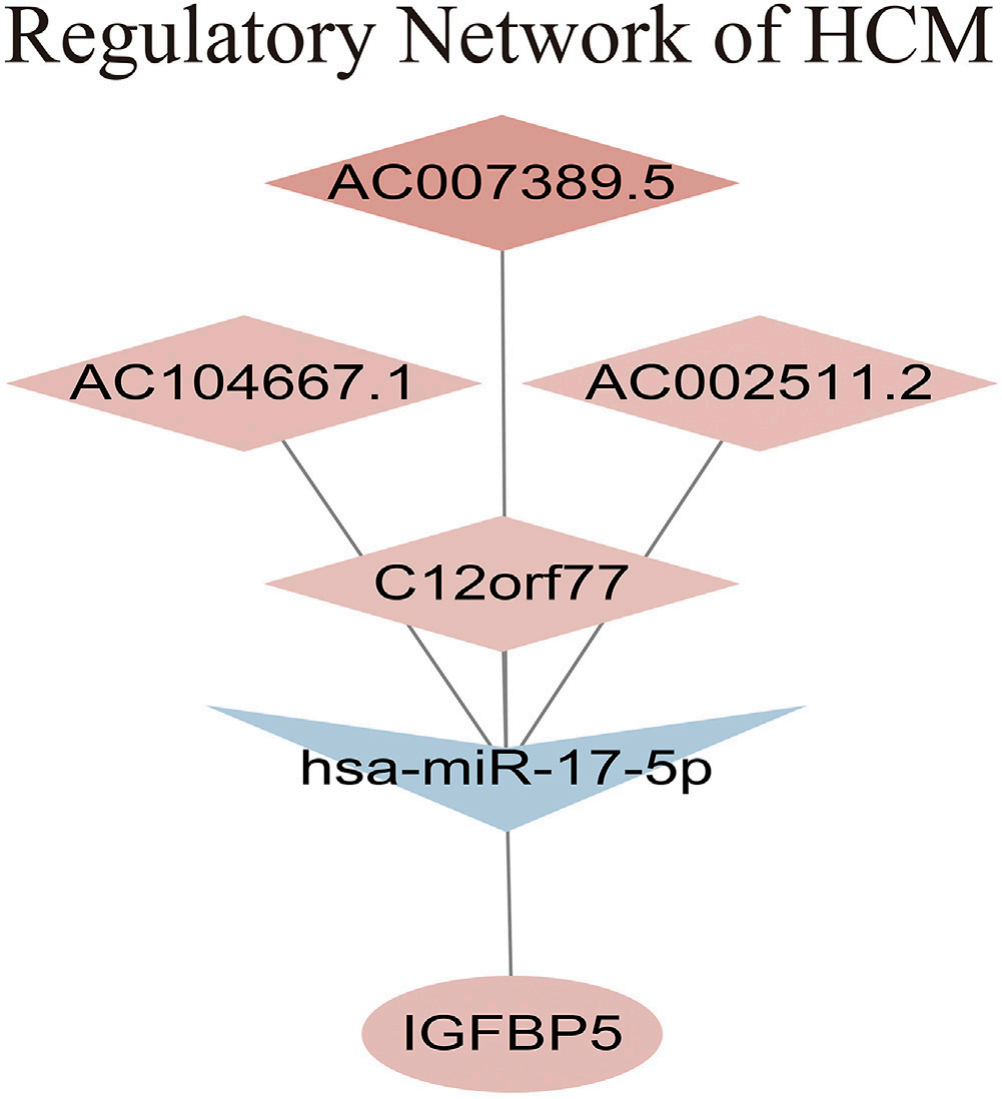
Regulatory network of HCM.

The intersection of genes between lncRNA-miRNA-mRNA network and above 2 key modules showed that gene *IGFBP5* was the clinically hub gene of HCM. A regulatory network of HCM was constructed as shown in Figure 6. The network indicated that the high expression of AC007389.5, AC104667.1 and AC002511.2 could down-regulate the expression of hsa-miR-17-5p in HCM tissue, which would ultimately up-regulate the expression of gene *IGFBP5*.

### Gene Set Enrichment Analysis of Clinically Hub Gene

**FIGURE 7:**
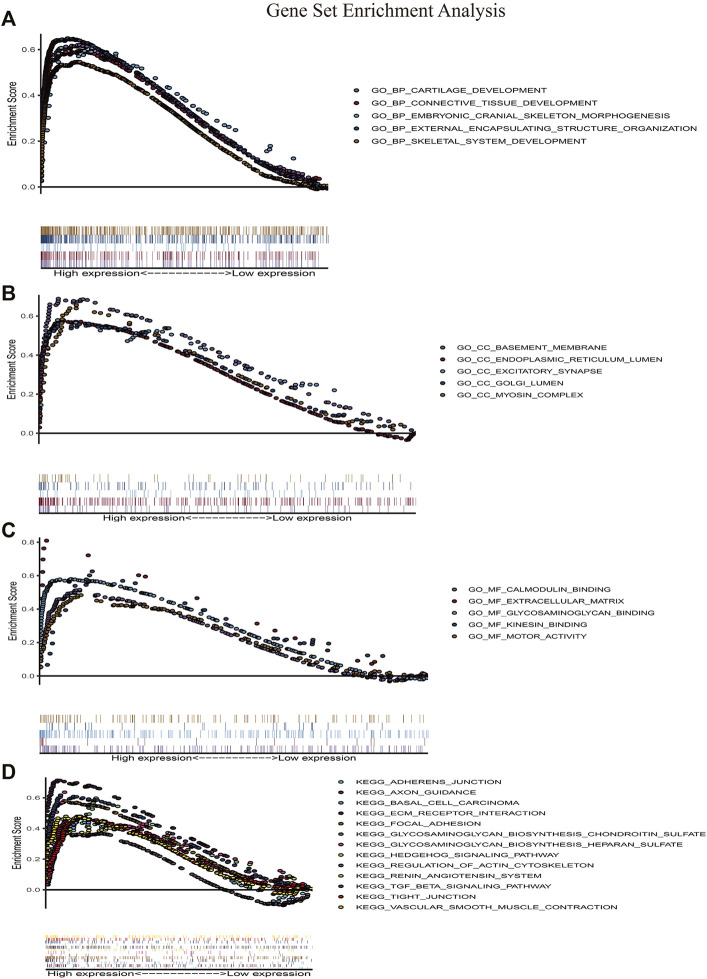
Gene set enrichment analysis. **(A–C)** Gene ontology enrichment analysis. **(D)** KEGG pathway enrichment analysis. HCM, hypertrophic cardiomyopathy.

We performed gene set enrichment analyses between IGFBP5 high expression group and *IGFBP5* low expression group. GO enrichment analysis showed that some biological processes were enriched in, for example, connective tissue development, external encapsulating structure (Figure 7A). GO enrichment analysis also revealed that cell components were related to basement membrane, endoplasmic reticulum lumen, excitatory synapse and myosin complex (Figure 7B). Besides, GO enrichment analysis also demonstrated that molecular functions were associated with calmodulin binding, extracellular matrix, glycosaminoglycan binding, kinesin binding and motor activity (Figure 7C). Furthermore, KEGG enrichment analysis indicated that the high expression of IGFBP5 might function through adherens junction, ECM receptor interaction, focal adhesion, hedgehog signaling pathway, regulation of actin cytoskeleton, renin angiotensin system, TGF β signaling pathway, tight junction, and vascular smooth muscle contraction (Figure 7D).

## Discussion

HCM is a cardiomyopathy that is inherited in an autosomal dominant pattern (Maron and Maron, 2013). It is an important cause of sudden death in young people (Shah, 2017; Maron, 2018). Although many researchers are committed to the pathogenesis of HCM currently, the mechanism of gene regulation is still poorly understood. This study constructed a ceRNA network involved in HCM via bioinformatic analysis on heart tissue. It will provide preference for diagnosis and treatment for HCM.

WGCNA is an important method for bioinformatics analysis. It builds a network according to the systematic gene expression so as to obtain genetic clusters that are functionally similar. Through WGCNA, we found that expression of genes in blue and black modules was negatively associated with development of HCM. GO functional analysis showed that the genes in black module contributed to muscle composition, myosin, actin, and fibronectin binding. Simultaneously, KEGG analysis demonstrated that the genes of this module were enriched in diastolic and contractile signaling pathways of cardiomyocytes. Moreover, genes in blue module were involved in cytokine chemotaxis, pathogen infection, inflammatory response, antigen presentation, and atherogenesis. This suggested that inflammation may play an important role in the progression of HCM.

In this study, differential genes were identified by Limma. Online search databases were used to pair lncRNA, miRNA, and mRNA, in order to establish an integrated ceRNA network. The constructed ceRNA network was composed of 11 lncRNAs, 4 miRNAs and 23 genes after successful pairing. The overexpression of lncRNA SNHG9 and AC010980 targeted at the expression of mRNA SLCTA5 and LRRC8A by up-regulating miR-184. Additionally, 3 miRNAs, including miR-876-3p, miR-17-5, and miR-139-5p, were targeted by 9 upstream lncRNAs. 21 genes were targeted by these 3 miRNAs. Several hub genes obtaining from Limma analysis have been reported to be involved in HCM in previous study, such as *RYR2*,*RBM20*, *XIRP2* (Helms et al., 2016; Okuda et al., 2018; Stanczyk et al., 2018; Fernlund et al., 2020). Importantly, *RyR2* is an important receptor that controls calcium channels of sarcoplasmic reticulum. Therefore, it is closely related to the systolic function of cardiomyocytes (Okuda et al., 2018; Stanczyk et al., 2018).

The genes of the above two modules were intersected with the ceRNA network. Gene *IGFBP5* was considered as the clinically hub gene of HCM. *IGFBP5* is a member of insulin-like growth factor binding proteins. By binding to insulin-like growth factor (IGF), *IGFBP5* plays an important role in cell differentiation and transcription (Amaar et al., 2002; Schedlich et al., 2007; Poreba and Durzynska, 2020). It can promote the proliferation and apoptosis of normal tissue cells and may further lead to tissue remodeling (Mano et al., 2017). Previous study discovered that *IGFBP5* takes part in the progression of atherosclerosis (Xu et al., 2004; Yang et al., 2021). *IGFBP5* promotes local inflammatory response of vascular endothelial cells, especially the involvement of macrophages. Macrophages propagates the development of plaque and promotes thrombosis, leading to the maintenance of inflammatory response (Moore et al., 2013). Meanwhile, *IGFBP5* is highly expressed in fibroblasts of lung and heart, taking part in promoting fibrosis of the tissues (S. E. Sureshbabu et al., 2009; Song et al., 2013). However, the role of *IGFBP5* in HCM has not been previously reported. Our study revealed that *IGFBP5* is correlated with the components of myosin complex. KEGG enrichment analysis showed that *IGFBP5* is enriched in regulation of actin cytoskeleton. As a result, *IGFBP5* might promote progression of HCM by influencing myosin and actin of myocardial cells. Of note, *IGFBP5* is also enriched in the renin angiotensin system, and the latter is thought to be an important regulatory mechanism leading to HCM (Musumeci et al., 2021). Further studies are expected to further uncover the molecular mechanisms involved.

Although we were able to establish a complete ceRNA network for HCM and identified potential pathogenic genes, several limitations of this study should be clarified. First of all, due to the difficulty in obtaining heart tissues, the experimental verification was missing. Subsequent experiments such as qPCR, Western blotting to support our conclusion will be required in the future. In addition, due to the limitations of data sources, we used multiple databases for the analysis. Heart tissues were treated in different experimental treatments, and the batch of effect could not be eliminated. Analysis of complete information from the same patient population will greatly promote construction of a convincing ceRNA network. Finally, the accuracy of the analysis results may be affected because the sample size for our research is relatively small.

In conclusion, we constructed a ceRNA network which is composed of 11 lncRNAs, 4 miRNAs, and 23 genes. It may play an important regulatory role in the progression of HCM. IGFBP5 might promote the progression of HCM, especially in tissue remodeling and regulation of actin cytoskeleton, which could be a potential biomarker for diagnosis of HCM. Its physiological role and detailed signaling pathway in HCM should be explored in the future.

## Data Availability

The datasets presented in this study can be found in online repositories. The names of the repository/repositories and accession number(s) can be found in the article/Supplementary Material.
