# Supplementary figures and images for: Susceptibility Modules and Genes in Hypertrophic Cardiomyopathy by WGCNA and ceRNA Network Analysis
Source: Front Cell Dev Biol. 2022 Feb 1;9:822465. doi: 10.3389/fcell.2021.822465 (PMC8844202; doi:10.3389/fcell.2021.822465)

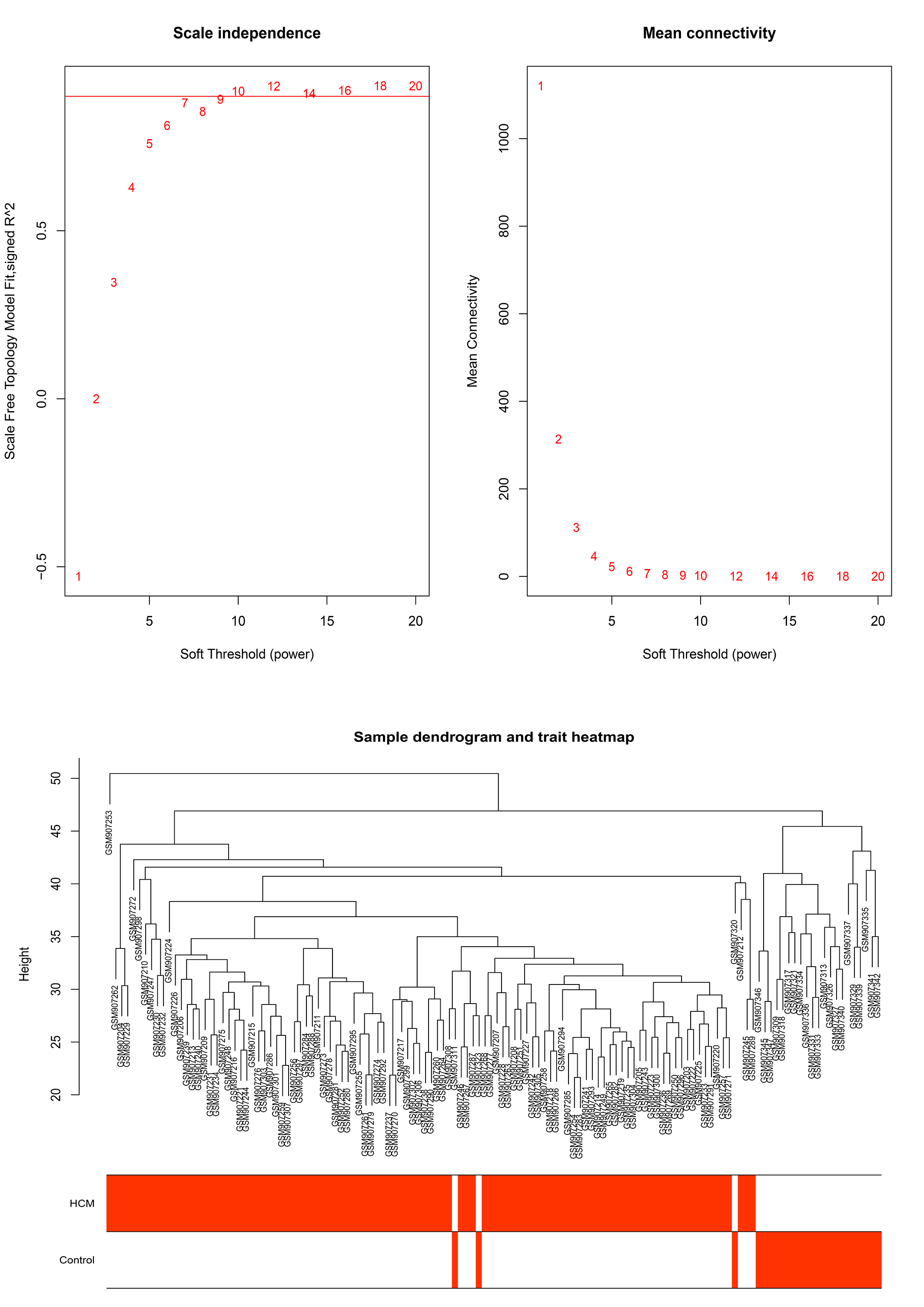

Supplement: Supplementary file 1 [file image1.tif]
